# First-Generation Versus Second-Generation Drug-Eluting Stents in Coronary Chronic Total Occlusions: Two-Year Results of a Multicenter Registry

**DOI:** 10.1371/journal.pone.0157549

**Published:** 2016-06-17

**Authors:** Jong-Hwa Ahn, Jeong Hoon Yang, Cheol Woong Yu, Je Sang Kim, Hyun Jong Lee, Rak Kyeong Choi, Tae Hoon Kim, Ho Joon Jang, Young Jin Choi, Young Moo Roh, Won-Heum Shim, Young Bin Song, Joo-Yong Hahn, Jin-Ho Choi, Sang Hoon Lee, Hyeon-Cheol Gwon, Seung-Hyuk Choi

**Affiliations:** 1 Division of Cardiology, Department of Medicine, Gyeongsang National University School of Medicine and Gyeongsang National University Changwon Hospital, Changwon, Republic of Korea; 2 Division of Cardiology, Department of Medicine, Cardiac and Vascular Center, Samsung Medical Center, Sungkyunkwan University School of Medicine, Seoul, Republic of Korea; 3 Department of Critical Care Medicine, Samsung Medical Center, Sungkyunkwan University School of Medicine, Seoul, Korea; 4 Division of Cardiology, Department of Medicine, Cardiovascular Center, Anam Hospital, Korea University Medical Center, Seoul, Republic of Korea; 5 Division of Cardiology, Department of Internal Medicine, Sejong General Hospital, Bucheon, Republic of Korea; St. Antonius Hospital, NETHERLANDS

## Abstract

**Background:**

Limited data are available regarding the long-term clinical outcomes of second-generation drug-eluting stents (DES) versus first-generation DES in patients with coronary chronic total occlusion (CTO) who undergo percutaneous coronary intervention (PCI). The aim of this study was to compare the clinical outcomes of second-generation DES with those of first-generation DES for the treatment of CTO.

**Methods and Results:**

Between March 2003 and February 2012, 1,006 consecutive patients with CTO who underwent successful PCI using either first-generation DES (n = 557) or second-generation DES (n = 449) were enrolled in a multicenter, observational registry. Propensity-score matching was also performed. The primary outcome was cardiac death over a 2-year follow-up period. No significant differences were observed between the two groups regarding the incidence of cardiac death (first-generation DES versus second-generation DES; 2.5% vs 2.0%; hazard ratio [HR]: 0.86; 95% confidence interval [CI]: 0.37 to 1.98; *p* = 0.72) or major adverse cardiac events (MACE, 11.8% vs 11.4%; HR: 1.00; 95% CI: 0.67 to 1.50; *p* = 0.99). After propensity score matching, the incidences of cardiac death (HR: 0.86; 95% CI: 0.35 to 2.06; *p* = 0.86) and MACE (HR: 0.93; 95% CI: 0.63 to 1.37; *p* = 0.71) were still similar in both groups. Furthermore, no significant differences were observed between sirolimus-eluting, paclitaxel-eluting, zotarolimus-eluting, and everolimus-eluting stents regarding the incidence of cardiac death or MACE.

**Conclusion:**

This study shows that the efficacy of second-generation DES is comparable to that of first-generation DES for treatment of CTO over 2 years of follow-up.

## Introduction

Percutaneous coronary intervention (PCI) of chronic total occlusion (CTO) lesions is a challenging procedure due to the difficulty in crossing the CTO and the high restenosis rates after PCI [1–4]. However, the success rate of treating CTO lesions has improved as cardiologists have gained experience in this technique and advances have been made in PCI technology. For instance, better outcomes of PCI of CTO lesions have been achieved with bare-metal stenting (BMS) compared with balloon angioplasty alone [1, 5, 6].

Drug-eluting stents (DES) were developed for enhanced stent durability compared with BMS by inhibiting in-stent neointimal hyperplasia. Sirolimus-eluting and paclitaxel-eluting stents (SES and PES), hereafter referred to as first-generation DES, are superior to BMS with respect to the in-stent restenosis rate and target lesion revascularization after CTO PCI [7–10]. However, everolimus-eluting and zotarolimus-eluting stents (EES and ZES), hereafter referred to as second-generation DES, have been found to be superior or comparable to first-generation DES for composite outcomes in non-CTO lesions [11–15]. In the context of CTO, a few studies have compared the impacts of second-generation DES on clinical outcomes with those of first-generation DES. However, these studies had relatively small sample sizes, short follow-up periods, and yielded contradictory results [16–19]. We therefore compared the long term outcomes of patients with CTO lesions who received second-generation DES with those of patients who received first-generation DES.

## Methods

### Study population

This study was conducted from prospective registries at two tertiary medical centers, Samsung Medical Center and Bucheon Sejong Hospital, in South Korea. Between March 2003 and February 2012, 2,659 consecutive patients were enrolled. The inclusion criteria for the registries were: 1) at least 1 CTO detected on a diagnostic coronary angiograph; and 2) symptomatic angina and/or a positive functional ischemia study. Exclusion criteria included: 1) previous coronary bypass grafting; 2) history of cardiogenic shock or cardiopulmonary resuscitation; and 3) ST-segment elevation acute myocardial infarction (MI) during the preceding 48 hours. A CTO lesion was defined as the obstruction of a native coronary artery with a Thrombolysis In Myocardial Infarction (TIMI) flow grade 0 and an estimated duration longer than 3 months (4). Duration was estimated based on the interval from the last episode of acute coronary syndrome (ACS). For patients with no history of ACS, duration was estimated from the first episode of exertional angina consistent with the location of the occlusion or previous coronary angiogram [18, 20, 21].

**Fig 1:**
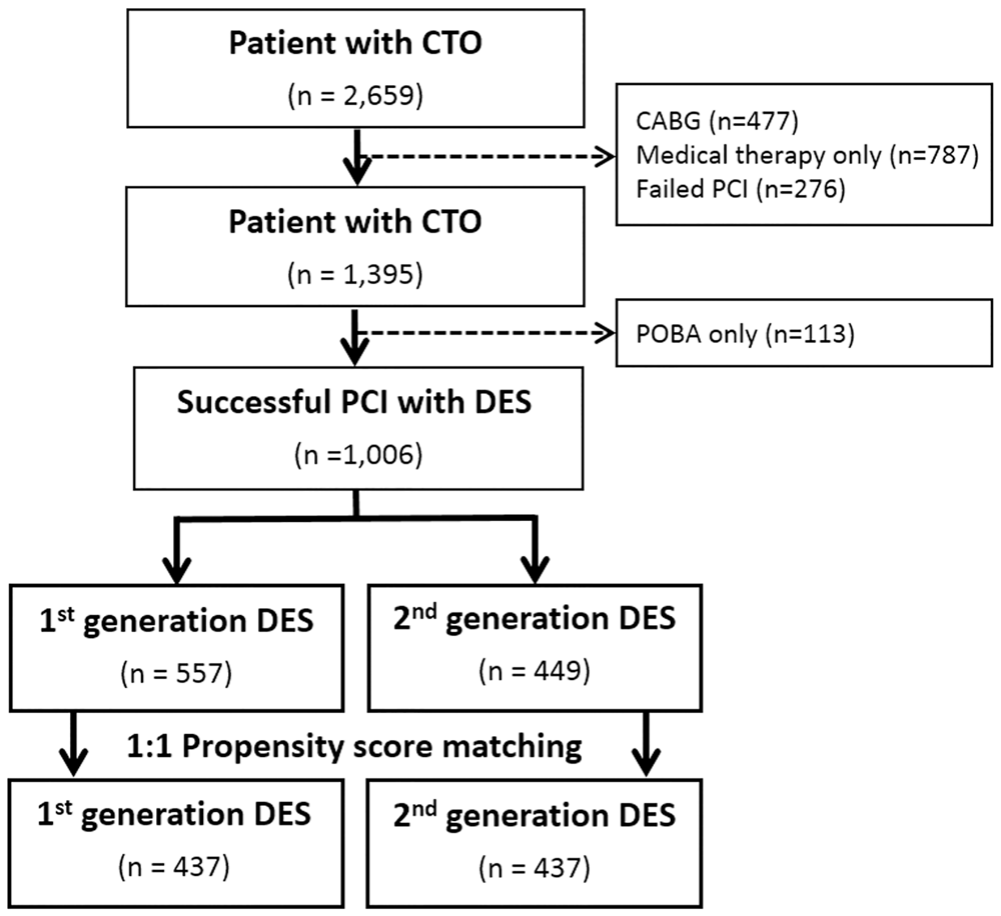
Profile of patient enrollment. CTO = chronic total occlusion, DES = drug-eluting stents, PCI = percutaneous coronary intervention.

Of the 2,659 patients included in the registry, 477 patients who underwent CABG and 787 patient who treated with medical therapy only were excluded. Of the patients who performed PCI, 1,196 patients (80.2%) underwent successful revascularization. Among them, 1,006 patients who underwent PCI with DES implantation and achieved angiographic success were finally included in this analysis (Fig 1).

### Data collection and follow-up

Experienced clinical research physicians and coordinators from an individual clinical research organization collected baseline clinical, angiographic and procedural characteristics from hospital charts or hospital databases according to prespecified definitions. Clinical follow-up of the registry after index coronary angiography was performed at 1, 3, 6, and 12 months, and every year thereafter. Collection of follow-up information was mainly conducted through review of inpatient and outpatient hospital charts by the clinical research coordinators, and additional follow-up information was collected through a telephone interview with patients and was confirmed with the Korean national database using a citizen registration number unique to each individual.

All baseline and procedural cine coronary angiograms were reviewed and quantitatively analyzed at the angiographic core laboratory (Cardiac and Vascular Center, Samsung Medical Center, Seoul, Korea) with an automated edge-detection system (Centricity CA 1000, GE, Waukesha, WI, USA) using standard definitions [22]. The extent of collateral flow was assessed according to the validated Rentrop classification scale [23].

### Percutaneous coronary intervention

Coronary interventions were performed according to a standard technique. All procedures and treatments, including periprocedural and postprocedural medication regimens, were performed according to current practice guidelines. All patients, regardless of stent type, received a 300 mg loading dose of aspirin and a 300 to 600 mg loading dose of clopidogrel before the coronary intervention, unless they had previously received these antiplatelet medications. DES were used without restriction; the duration of dual antiplatelet therapy and the use of glycoprotein IIb/IIIa antagonists was left to the discretion of the operator.

### Study outcomes and definitions

The primary outcome was cardiac death over a 2-year follow-up period. The secondary outcomes were all-cause death, MI, repeat revascularization, and major adverse cardiac events (MACEs). Repeat revascularization included target vessel revascularization (TVR) and non-TVR treated with PCI or CABG. MACEs included cardiac death, MI, and repeat revascularization. All deaths were considered to be of cardiac origin unless a definite noncardiac cause could be established. MI was defined as an elevation of the creatine kinase-MB fraction or the troponin-T/troponin-I level greater than the upper normal limit with concomitant ischemic symptoms or electrocardiographic findings indicative of ischemia [24]. Periprocedural MI was defined as an elevation of the creatine kinase-MB fraction ≥ 3 times the upper limit of normal after the index procedure [25]. Periprocedural MI was not included in this definition of MI.

### Statistical analysis

Continuous variables are expressed as means ± SDs, and categorical variables are presented as absolute numbers and proportions (%). Overall comparisons between groups were performed by Student’s *t*-test for continuous variables and by the chi-square test or Fisher’s exact test when the Cochran rule was not met for categorical variables. The Cox proportional hazards model was used to estimate the hazard ratio (HR) and 95% confidence interval (CI) for clinical outcomes between two groups. Cumulative event rates were estimated by the Kaplan-Meier method, and treatment effects were assessed using stratified log-rank statistics. We also adjusted for differences in clinical and angiographic characteristics by performing propensity score matching. The “psmatching” custom dialogue was used in conjunction with SPSS version 21 (IBM, Armonk, NY, USA). The psmatching program performs all analyses in R (R foundation for Statistical Computing, Vienna, Austria) though the SPSS R-Plugin (version 2.14.2). Using the propensity score matching method, we created 557 matched pairs of patients. The adequacy of propensity matching was calculated by the overall balance test (chi-square = 4.79, df = 22.00, and *p* = 0.98). All tests were 2-tailed, and *p* values < 0.05 were considered to indicate statistical significance.

### Ethical approval

The design of the study was approved by Institutional Review Board at Samsung Medical Center and Bucheon Sejong Hospital. The institutional review boards approved this study and waived the requirement for informed consent. All patients’ records/information were anonymized and de-identified prior to analysis.

## Results

### Patient characteristics

Of the 2,659 patients included in the registry, 1,006 patients received only first-generation (n = 557, first-generation group) or second-generation DES (n = 449, second-generation group). In the first-generation group, 59.4% of the recipients received SES (n = 331, SES group), whereas 40.6% of the recipients received PES (n = 226, PES group). In the second-generation group, 62.4% of the recipients received EES (n = 280, SES group) and 36.6% of the recipients received ZES (n = 168, ZES group).

The patients in the first-generation group were more likely to be male and less likely to have had a history of dyslipidemia or ACS compared with the patients in the second-generation group (Table 1). As shown in Table 2, patients in the first-generation group also had a significantly higher prevalence of abrupt stumps and longer total stent lengths, but lower prevalences of Rentrop grade 3 collateral flow and proximal to mid CTO lesions compared with patients in the second-generation group. After performing propensity score matching for the entire population, all 437 patients in the first-generation group were matched with patients in the second-generation group. No significant differences were observed between the first-generation DES and second-generation DES groups regarding baseline clinical, angiographic, or procedural characteristics (Tables 1 and 2).

**Table 1:** Baseline characteristics.

Variables	Total population				Propensity-Matched population		
	Total (n = 1,006)	1st generation (n = 557)	2nd generation (n = 449)	P value	1st generation (n = 437)	2nd generation (n = 437)	P value
**Age (yrs)**	62.5 (± 11.2)	62.3 (± 10.9)	62.8 (± 11.6)	0.48	62.4 (± 11.0)	62.7 (± 11.7)	0.61
**Male**	784 (77.9%)	448 (80.4%)	336 (74.8%)	**0.03**	339 (77.6%)	331 (75.7%)	0.52
**Diabetes**	408 (40.6%)	231 (41.5%)	177 (39.4%)	0.51	181 (41.4%)	173 (39.6%)	0.58
**Hypertension**	626 (62.2%)	338 (60.7%)	288 (64.1%)	0.26	281 (64.3%)	283 (64.8%)	0.89
**Dyslipidemia**	452 (44.9%)	231 (41.5%)	221 (49.2%)	**0.01**	197 (45.1%)	212 (48.5%)	0.31
**Current smoker**	313 (31.1%)	171 (30.7%)	142 (31.6%)	0.75	138 (31.6%)	138 (31.6%)	1.00
**Chronic kidney disease**	59 (5.9%)	28 (5.0%)	31 (6.9%)	0.21	25 (5.7%)	30 (6.9%)	0.49
**ACS**	299 (29.7%)	150 (26.9%)	149 (33.2%)	**0.03**	130 (29.7%)	139 (31.8%)	0.51
**CVA**	32 (7.9%)	7 (9.0%)	25 (7.7%)	0.70	30 (6.9%)	26 (5.9%)	0.58
**Previous PCI**	210 (20.9%)	119 (21.4%)	91 (20.3%)	0.67	93 (21.3%)	91 (20.8%)	0.87
**LVEF (%)**	57.5 (± 12.2)	57.5 (± 11.9)	57.4 (± 12.4)	0.862	57.9 (± 11.7)	57.6 (± 12.4)	0.65
**Discharge medication**							
**Statins**	773 (76.8%)	421 (75.6%)	352 (78.4%)	0.29	335 (76.7%)	344 (78.7%)	0.47
**Beta-blocker**	599 (59.5%)	345 (61.9%)	254 (56.6%)	0.09	262 (60.0%)	248 (56.8%)	0.34
**ACE inhibitors or ARB**	608 (60.4%)	340 (61.0%)	268 (59.7%)	0.66	263 (60.2%)	260 (59.5%)	0.84

Values are mean ± SD or n (%). ACE = angiotensin-converting enzyme; ACS = acute coronary syndrome; ARB = angiotensin receptor blocker; CVA = cerebrovascular accident; LVEF = left ventricular ejection fraction; PCI = percutaneous coronary intervention.

**Table 2:** Angiographic characteristics.

Angiographic parameters	Total population				Propensity-Matched population		
	Total (n = 1,006)	1st generation (n = 557)	2nd generation (n = 449)	P value	1st generation (n = 437)	2nd generation (n = 437)	P value
**Abrupt stump**	360 (35.8%)	216 (38.8%)	144 (32.1%)	**0.03**	148 (33.9%)	141 (32.3%)	0.62
**Bridge collaterals**	256 (25.4%)	149 (26.8%)	107 (23.8%)	0.29	113 (25.9%)	104 (23.8%)	0.48
**Calcification**	123 (12.2%)	70 (12.6%)	53 (11.8%)	0.71	58 (13.3%)	49 (11.2%)	0.35
**CTO location**							
**Left main**	3 (0.3%)	0 (0.0%)	3 (0.7%)	0.05	0 (0.0%)	0 (0.0%)	-
**LAD**	413 (41.1%)	243 (43.6%)	170 (37.9%)	0.07	173 (39.6%)	165 (37.8%)	0.58
**LCx**	294 (29.2%)	149 (26.8%)	145 (32.3%)	0.06	135 (30.9%)	141 (32.3%)	0.66
**RCA**	419 (41.7%)	234 (42.0%)	185 (41.2%)	0.78	179 (41.0%)	179 (41.0%)	1.00
**Rentrop grade 3**	302 (30.1%)	144 (25.9%)	158 (35.2%)	**0.001**	138 (31.6%)	149 (34.1%)	0.43
**Mutivessel disease**	705 (70.1%)	394 (70.7%)	311 (69.3%)	0.61	313 (71.6%)	303 (69.3%)	0.46
**Maximal stent diameter (mm)**	3.0 (± 0.4)	3.0 (± 0.4)	3.0 (± 0.5)	0.46	3.0 (± 0.4)	3.0 (± 0.5)	0.94
**Total stent length (mm)**	33.6 (± 15.4)	35.0 (± 15.3)	31.9 (± 15.4.4)	**0.002**	33.1 (± 13.5)	31.7 (± 14.7)	0.15
Proximal to mid CTO*	733 (72.9%)	422 (75.8%)	311 (69.3%)	**0.02**	315 (72.1%)	302 (69.1%)	0.33

Values are n (%) or mean ± SD. CTO = coronary chronic total occlusion; LAD = left anterior descending artery; LCx = left circumflex artery; RCA = right coronary artery

*“CTO of the proximal to middle portions of the vessel” has been abbreviated as “Proximal to mid CTO.”

### Clinical outcomes

The median follow-up duration for all surviving patients was 1,265 days [interquartile range (IQR): 764–1,968 days]. As expected, the median follow-up duration was shorter for patients treated with second-generation DES [median 881 days (IQR: 535–1,255 days)] compared with patients treated with first-generation DES [median 1,779 days (IQR: 1,186–2,330 days)]. The disparity in follow-up duration between the two groups, which was unavoidable because of the later development of second-generation DES, was accounted for in the analysis by considering only the first 24 months after PCI.

**Fig 2:**
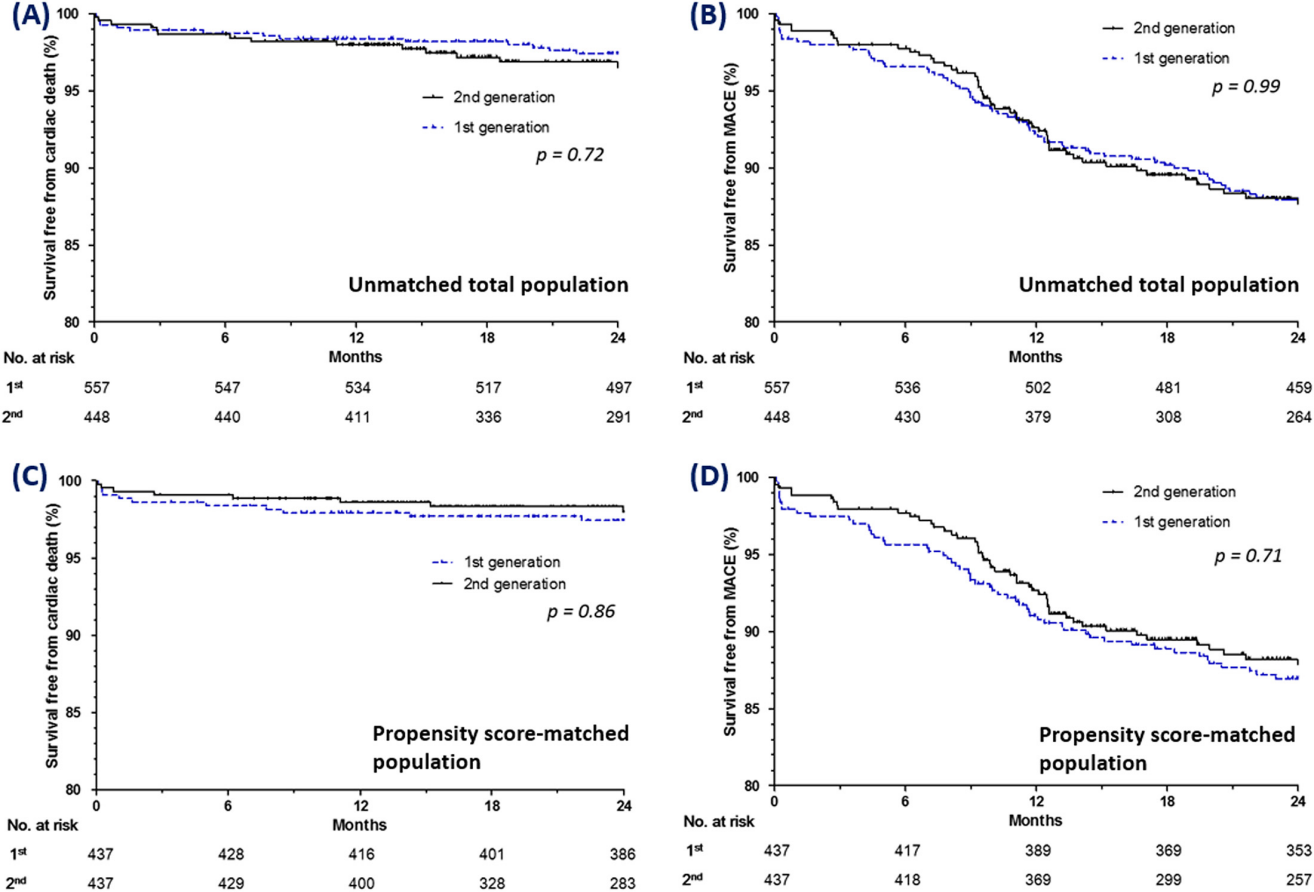
Kaplan-Meier curves for clinical outcomes in all the patients (A, B) and the propensity-matched patients (C, D). Kaplan-Meier curves for cardiac death and MACE in the patients treated with first-generation and second generation drug-eluting stents. MACE = major adverse cardiac event.

After 2 years of follow-up, 14 cardiac deaths had occurred in the first-generation group and 9 cardiac deaths had occurred in the second-generation group (2.5% versus 2.0%; HR: 0.86; 95% CI: 0.37 to 1.98; *p* = 0.72). The rates of all-cause death, MI, repeat revascularization, and MACE were also not different between the two groups (Table 3). Kaplan-Meier curves for survival free from cardiac death and survival free from MACE after 2 years of follow-up are shown for both groups in Fig 2A and 2B, respectively. After 1:1 propensity score matching, the clinical outcomes during follow-up were not significantly different between the two groups (Table 3, Fig 2C and 2D).

**Table 3:** Clinical outcomes.

	Total population					Propensity-Matched population			
	Total (n = 1,006)	1st generation (n = 557)	2nd generation (n = 449)	HR (95% CI)	P value	1st generation (n = 437)	2nd generation (n = 437)	HR (95% CI)	P value
**Cardiac death**	23 (2.3%)	14 (2.5%)	9 (2.0%)	0.86 (0.37–1.98)	0.72	11 (2.5%)	9 (2.1%)	0.86 (0.35–2.06)	0.86
**All-cause death**	42 (4.2%)	27 (4.8%)	15 (3.3%)	0.76 (0.40–1.42)	0.39	22 (5.0%)	15 (3.4%)	0.74 (0.38–1.42)	0.36
**Myocardial infarction**	10 (1.0%)	7 (1.3%)	3 (0.7%)	0.55 (0.14–2.13)	0.39	6 (1.4%)	3 (0.7%)	0.51 (0.13–2.02)	0.33
**Repeat revascularization**	98 (9.7%)	56 (10.1%)	42 (9.4%)	1.01 (0.70–1.47)	0.95	48 (11.0%)	40 (9.2%)	0.89 (0.58–1.35)	0.89
MACE*	117 (11.6%)	66 (11.8%)	51 (11.4%)	1.00 (0.67–1.50)	0.99	56 (12.8%)	49 (11.2%)	0.93 (0.63–1.37)	0.71

Values are n (%).CI = confidence interval; HR = hazard ratio; MI = myocardial infarction.

*Major adverse cardiac events (MACE) included cardiac death, MI, and repeat revascularization (included target vessel revascularization-PCI, non–target vessel revascularization-PCI, or coronary artery bypass grafting).

### Subgroup analysis

**Fig 3:**
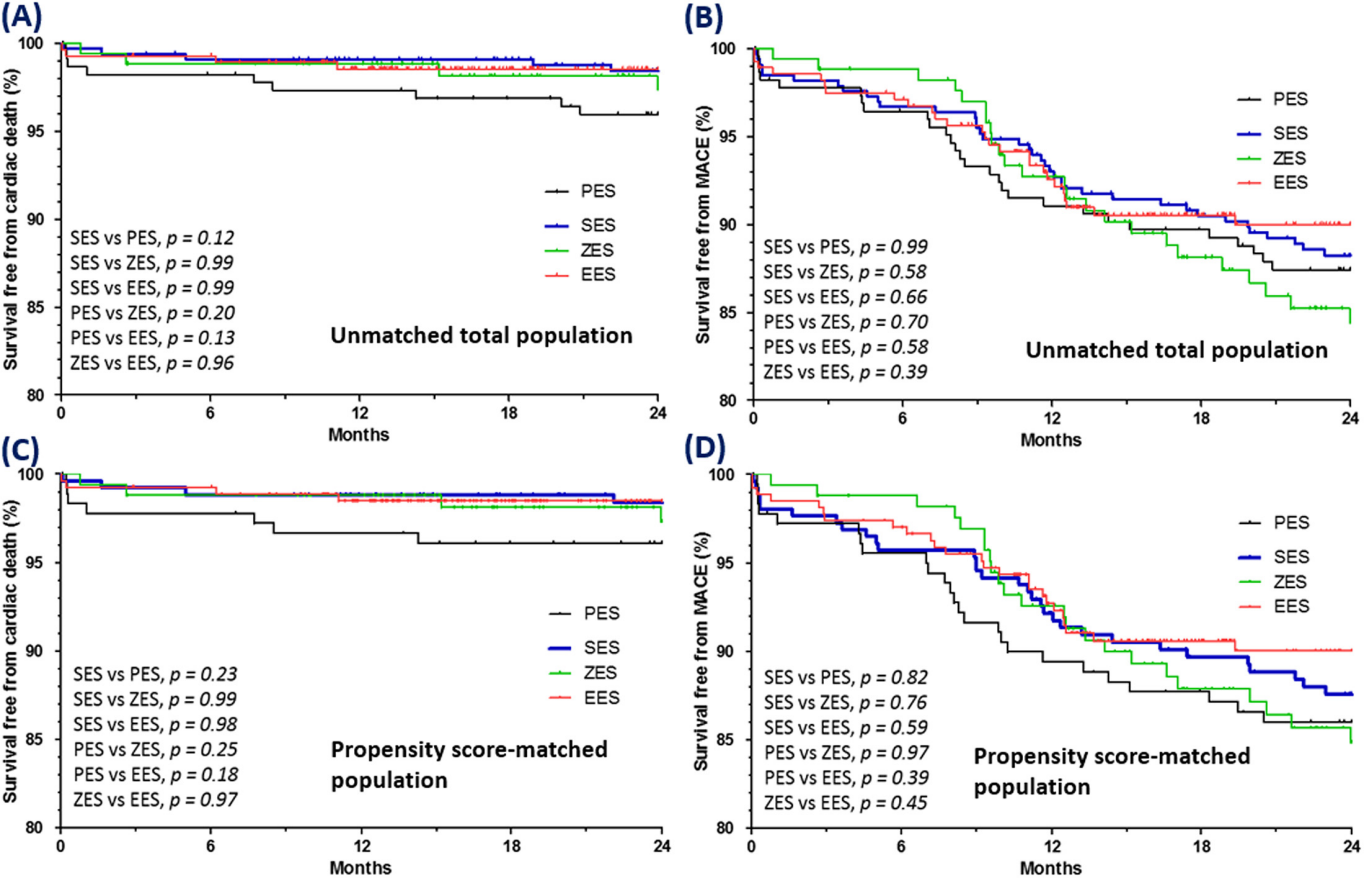
Kaplan-Meier curves for cardiac death and MACE according to DES subgroup for all patients (A, B) and propensity-matched patients (C, D). MACE = major adverse cardiac event; DES = drug-eluting stent; SES = sirolimus-eluting stent; PES = paclitaxel-eluting stent; EES = everolimus-eluting stent; ZES = zotarolimus-eluting stent.

We next examined the following four subgroups for differences: SES, PES, EES, and ZES. After 2 years, no significant between-group differences were observed regarding the rates of cardiac death, MI, repeat revascularization, or MACE. Kaplan-Meier curves for survival free from cardiac death and survival free from MACE after two years of follow-up are shown for each of the four subgroups in Fig 3.

**Fig 4:**
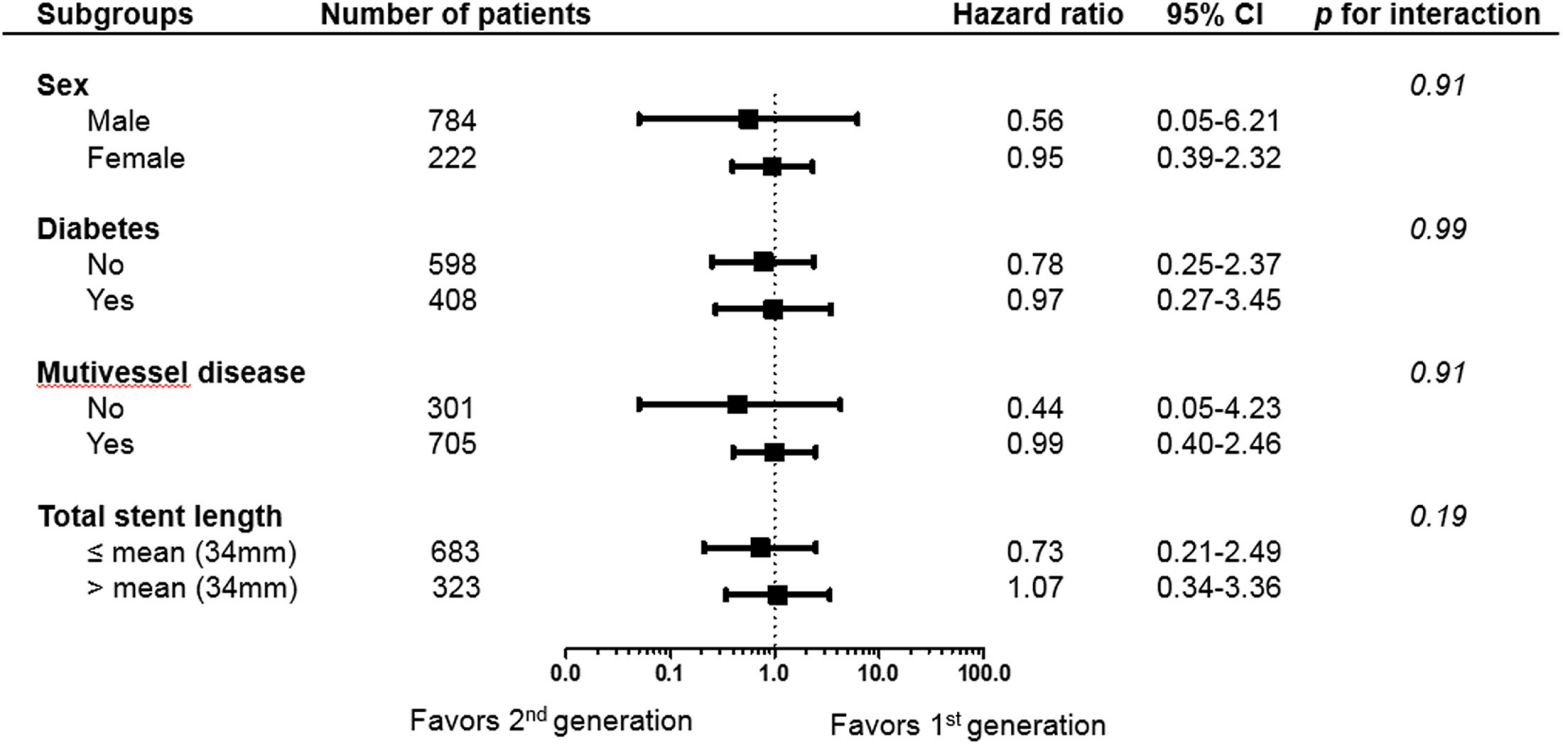
Comparative unadjusted hazard ratios of cardiac death for the DES subgroups out of all the patients between first-generation and second-generation drug-eluting stents. CI = confidence interval.

Finally, we calculated the unadjusted HR for cardiac death in various subgroups to determine whether the outcomes according to second-generation DES (vs. first-generation DES) observed in the overall population were consistent in these subgroups (Fig 4). No significant interactions were observed between the use of second-generation DES and cardiac death in any of the subgroups.

## Discussion

In this study, we demonstrated that first-generation and second-generation DES are similarly effective in the context of CTO lesions. No differences were found between the two groups regarding the overall incidence of cardiac death or the overall incidence of MACE, despite the mid-term (two years) period of observation. After propensity score matching of all the patients, the incidences of cardiac death and MACE in the two groups were still comparable. Subgroup analyses according to DES type (SES, PES, EES, and ZES) also revealed similar incidences of cardiac death and MACE. In fact, previous meta-analyses substantially differ from the current one. In the meta-analysis by Lanka et al. [26] only 5 studies were collected reporting 1,174 patients. Although a significant benefit on all cause death in patients assigned to second-generation DES was reported, the effects on cardiac death was not analyzed.

EES have been found to be superior to PES [11, 27–29] and comparable to SES [30, 31] with respect to cardiac death, MI, stent thrombosis, and revascularization in randomized trials and prospective cohort studies of general populations. ZES have also been found to be superior to PES [15, 32] and comparable to SES [32, 33] with respect to death rate and MI in randomized trials of general populations. Although some of these studies included patients with CTO lesions, few such analyses have been carried out.

Several randomized and nonrandomized studies have compared outcomes between first-generation and second-generation DES after PCI in CTO lesions. Valenti et al [18] examined 588 patients with CTO and found that second-generation DES (EES) were associated with reduced cardiac death and MACE (11.6% vs. 22.4%, *p <* 0.01) compared with first-generation DES (SES and PES). Although this study had a relatively large number of patients, the registry was from a single center and was not randomized; moreover, the follow-up duration was only 12 months. In contrast to this study, Moreno et al [16], Park et al [17], and Almalia et al [19] observed no significant differences in the rates of cardiac death and MACE between first-generation and second-generation DES. Moreno et al performed a prospective randomized study of 207 patients with CTO and found that EES was comparable to SES with respect to cardiac death (0.9% vs. 2.0%, *p* = 0.52) and MACE (11.3% vs. 15.8%, *p* = 0.34). Park et al also performed a prospective randomized study of 160 patients with CTO and found that ZES was comparable to SES with respect to cardiac death (1.3% vs. 2.5%, *p* = 0.56) and repeat revascularization (10% vs. 17.5%, *p* = 0.17). Although these two studies were prospective randomized studies, their sample sizes were relatively small and the follow-up duration was only 12 months. Therefore, we compared the impacts of second-generation and first-generation DES on clinical outcomes in patients with CTO from a large-scale, multicenter registry with a relatively long-term follow-up duration.

Second-generation DES have several advantages for PCI for CTO lesions. First, stent platforms of cobalt or platinum-chromium alloys are thinner and more deliverable than the platforms used in first-generation DES. This improved flexibility and deliverability might increase the success rate in treating CTO lesions. Moreover, second-generation DES produce a weaker inflammatory response and stimulate more rapid vessel re-endothelialization. Despite these improvements of second-generation DES, similar incidences of cardiac death, MI, and MACE have been observed in patients with bifurcated lesions [34] and ACS [35] treated with second-generation DES vs first-generation DES. Overall, the results from our CTO registry are consistent with previous 12-month follow-up prospective randomized CTO studies [16, 17] in that second-generation DES do not seem to confer any major advantages to clinical outcomes such as cardiac death, MI, or repeat revascularization.

Our study did have several limitations. First, the study design was nonrandomized due to the nature of the registry data, which means that confounding factors may have affected the results. Second, we lacked comprehensive data regarding possible alterations of medical therapies over the follow-up period. Third, we did not routinely perform angiographic follow-up examination on our patients; thus, angiographic adverse events may have been underestimated. Fourth, although the vital status of all patients, including those lost to follow-up, was confirmed with the Korean national database using a citizen registration number that is unique to each individual, we cannot exclude the possibility of under-reporting of clinical outcomes other than death such as nonfatal MI and stent thrombosis. Fifth, our attempt to mitigate the unavoidable disparity in follow-up duration due to the time lag between the development of first-generation and second-generation DES limited our outcome analysis to 2 years. Furthermore, newer specialized revascularization devices for the treatment of CTO lesions may have been used more often in the second-generation DES group, which may have led to overestimation or underestimation of MACEs. Finally, the present analysis included patients who were treated over a long period of time. During this time, changes in PCI strategies may have impacted the clinical outcomes, irrespective of the stent type used.

## Conclusion

Our results suggest that the efficacy of second-generation DES is similar to that of first-generation DES for patients with CTO who undergo PCI, at least over a 2 year follow-up period.

## Supporting Information

S1 Dataset De-identified minimal dataset. It includes data of CTO patients with file format of Microsoft Excel.
(XLSX)
